# Sorting the confusion about the numerous versions of the Barratt Impulsiveness Scale

**DOI:** 10.1007/s10072-023-06907-5

**Published:** 2023-06-15

**Authors:** Adrian Meule

**Affiliations:** 1 Department of Psychiatry and Psychotherapy, University Hospital of the LMU Munich, Munich, Germany; 2 Schoen Clinic Roseneck, Prien am Chiemsee, Germany

The Barratt Impulsiveness Scale (BIS) is one of the most widely used self-report questionnaires for the assessment of impulsivity as a trait. The original BIS was developed by Ernest S. Barratt in 1959 [1]. Since then, the scale has been revised several times and the current version is the BIS–11 [1]. Note that the number 11 does not refer to the number of items included in the questionnaire but to the number of revisions, that is, the BIS–11 is the 11^th^ version of the BIS, which has 30 items. There is also a provisional version of the BIS–11—the BIS–11A—that was distributed during development of the BIS–11 and that has been more widely disseminated and used than was intended. As the BIS–11 and BIS–11A only share 24 items in common, it has been recommended that the BIS–11A should not be used [1].

Complicating matters, abbreviated versions of the BIS–11 have been developed in the past decades. These include, for example, the BIS–Brief, which includes 8 of the BIS–11’s 30 items [2]. Another popular short form of the BIS–11 is the BIS–15 developed by Spinella [3]. In contrast to the BIS–11, the number 15 refers to the number of items included in the questionnaire, that is, the BIS–15 contains 15 items of the 30-item BIS–11. As of this writing (June 2023), the article by Spinella [3] has been cited more than 600 times according to Google Scholar and translated versions of the BIS–15 have been used in several other languages such as German, French, Spanish, Thai, and Kannada (see [4] for an overview).

Recently, Maggi and colleagues [5] reported on the development of a short version of the Italian BIS–11 in this journal. Unfortunately, they named this short form of the BIS–11 the BIS–15, obviously being unaware that a version with such a name already exists (i.e., the article by Spinella [3] or any other article about the BIS–15 is not cited in that article). This is particularly unfortunate as the items of Spinella’s BIS–15 and Maggi et al.’s BIS–15 are not identical. While 12 items of the two versions overlap, Spinella’s BIS–15 include the items “I plan for the future.”, “I am restless at lectures or talks.”, and “I squirm at plays or lectures.” while Maggi et al.’s BIS–15 include the items “I am happy-go-lucky.”, “I am self-controlled.”, and “I spend or charge more than I earn.”.

While I appreciate the effort of Maggi and colleagues [5] to develop a short form that is more psychometrically sound than the Italian BIS–11 (and it is quite remarkable that this lead to a selection of items closely similar to the English items selected by Spinella [3]), it further adds to the confusion that there are several versions of the BIS in general and several short versions of the BIS–11 in particular. Thus, Maggi and colleagues or other researchers who use the Italian BIS–15 may consider referring to it with a different name to avoid confusing this scale with Spinella’s BIS–15. I further suggest that Maggi and colleagues may reanalyze their data and examine if using the BIS–Brief’s 8 items or the original BIS–15’s 15 items may also represent psychometrically sound short versions of the Italian BIS–11. If so, using these versions will facilitate comparability across studies and reduce confusion about the different versions of the BIS.

## Data Availability

Not applicable.
